# Book Reviews

**Published:** 1829-11

**Authors:** 


### CRITICAL ANALYSES.

Quæ laudanda forent, et quæ culpanda, vicissim

Illa, prius, cretâ; mox hæc, carbone, *notamus*.—Persius.

*Pathological and Practical Researches on Diseases of the Brain and Spinal Cord.* By John Abercrombie, m.d. Fellow of the Royal College of Physicians of Edinburgh, &c. and first Physician to his Majesty in Scotland. *Second Edition, enlarged.* —8vo. pp. 476. Waugh and Innes, Edinburgh, 1829.

As we have always expressed a very favorable opinion of Dr. Abercrombie's pathological researches, we are, of course, gratified to find that a second edition of the present work is so soon required. Unlike many writers on pathology, Dr. Abercrombie has no exclusive doctrines to support which can interfere with the unbiassed history of disease, as he has witnessed it at the bedside of the patient. "He may indulge in conjectures, but these he keeps entirely distinct from the facts upon which they are founded;" and, by having strictly adhered to this principle, he has given to the profession a more interesting and practically useful body of evidence on diseases of the brain, stomach, and other abdominal viscera, than has been furnished by any other modern writer.

We have before noticed the first edition of the present work, and shall now confine ourselves to an analysis of the additional matter contained in the second. With the general divisions and arrangement of the work we must presume our readers to be acquainted.

Since the publication of the first edition, Dr. Abercrombie has seen several examples of a dangerous modification of Meningitis, which does not usually admit of very active treatment. General bleeding is not borne well; and the treatment must commonly be confined to topical bleeding, with purgatives, antimonials, and the powerful application of cold to the head. In such cases the cause of death is obscure. "It seems in general to be a sudden sinking of the vital powers supervening upon the high excitement, without any of the actual results of inflammation." In these instances Dr. A. has lately been induced to adopt a different mode of treatment from that which he originally recommended; and, as an example of the occasional efficacy of it, (for he does not at present venture upon any general conclusions,) the following case is related.

"A lady, aged about thirty-eight, was recovering from her eleventh accouchement, when, at the end of a fortnight, she became affected with a deep-seated hard swelling in the right side of the pelvis, which was tender to the touch, and was accompanied by a considerable degree of fever. After repeated topical bleeding and other remedies, the febrile state subsided, the swelling lost its tenderness, and seemed to be gradually diminishing in size; but its progress was very slow, and, after three or four weeks, she was still confined to bed, and suffering a great deal of uneasiness: her pulse was now calm, but she was considerably reduced in strength. At this time she became, one day, alarmed and agitated by some family occurrence, and immediately began to talk wildly and incoherently; and, after a restless night, was found, next day, in a state of the highest excitement, talking incessantly, screaming, and struggling, with a wild expression of countenance, and a small rapid pulse. She was treated by topical bleeding, laxatives, cold applications to the head, &c., but with little or no benefit; and, on visiting her on the following day, I found her sitting up in bed, with a look of extreme wildness, both her hands in constant motion, talking incessantly and wildly; and I learnt that she had not ceased talking for one instant for the last twelve hours. Her pulse was now rapid and feeble, and her countenance expressive of exhaustion. In consultation with a highly intelligent friend who had charge of the case, I mentioned my experience of the fatal nature of the affection, and proposed to make trial of treatment by stimulants. A glass of wine was accordingly given, with evident abatement of the symptoms; and it was ordered to be repeated every hour. At the end of the fourth hour, she was perfectly composed and rational, her pulse about ninety, and of good strength; and from this time there was no return of the symptoms. The tumor in the right side increased in size, suppurated, was opened, and healed favorably. From this time she continued in perfect health, and has since passed through another accouchement in the most favorable manner.

"This case I have given as another example of this interesting affection. I have employed the same mode of treatment, with similar benefit, in several other cases, both of males and females. The chief difficulty is in deciding upon the particular cases to which the stimulating treatment is applicable. They appear to be those in which the excitement is accompanied by a small and rapid pulse, and an expression of paleness and exhaustion. When these characters are present, however violent the excitement may be, I have not been deterred from the practice, and, in a considerable number of instances, have found much reason to be satisfied with it. I have tried it, but without the same benefit, in some of the common cases of insanity, accompanied by paleness and bodily weakness, but with a natural pulse. When there is frequent and strong pulse, with flushing and other marks of increased vascular action, it would of course be injurious." (P. 67.)

It is difficult, nay perhaps impossible, even with the utmost fidelity of description, to convey to the minds of others a precise knowledge of the nature of any particular case: but, unless we are mistaken, we have seen cases similar to the above, in which, when bleeding, purgatives, and cold have been employed, from a conviction that the disease was purely inflammatory, and the patient has still remained restless and loquacious, with a small pulse, pale countenance, and appearances of exhaustion, almost immediate relief has been derived from the use of opiates. Of course, much practical tact and experience will be required to enable the practitioner to determine when either stimuli or opiates may be advantageously, or even safely, prescribed in such instances.

The following cases of softening of the brain are particularly interesting.

" A gentleman, aged twenty-six, of a plethoric habit, had suffered occasionally for two or three years from headach and vertigo, which were always relieved by depletion. On 12th April, 1827, while walking out, he was seized with confusion and giddiness, embarrassed speech, and a considerable degree of paralysis of the right leg. He was rather pale; his pulse was seventy, and soft; and he did not complain of any headach. The usual treatment was adopted with activity by Dr. Combe, of Leith, without much relief. On the contrary, after several days he began to complain of acute headach, accompanied by vomiting and hiccup; and the other symptoms continued nearly as before, his speech being laboured and slow, and his memory very defective. After some weeks those symptoms subsided, so that he was able to walk out; but the headach continued, with frequent vomiting. The pain was chiefly referred to the left side of the head, sometimes to the occiput, and there was occasional numbness of the right arm. When I saw him, along with Dr. Combe and Dr. Kelly, in July, his chief complaint was of frequent and irregular attacks of vomiting, occurring daily, or repeatedly during the day. It came on very suddenly, without previous nausea, and he was often awakened in the night by the sudden attack of vomiting. He had now a pale sickly look; there was no paralytic affection, and little complaint of headach, though he still had occasional uneasiness in the head, sometimes referred to one part of it, and sometimes to another. When he did refer it to a particular part as the principal seat of the pain, it was either the left temple or the occiput. But the headach at this time was slight and transient, and the symptoms in the stomach were so much the more prominent that it was a matter of much doubt whether there was now any fixed disease of the head. The vomiting was much relieved by the oxyd of bismuth, so that he was free from it for several days. But it soon returned and went on as before, with increasing debility, great listlessness, and bad appetite; pulse little affected. He had now a peculiar unsteadiness of his limbs, so that, on first getting up into a standing posture, he staggered very much, and required some time and attention to steady himself. When he accomplished this, he walked with tolerable firmness. The symptoms went on in this manner till the 27th of October, when he was suddenly seized with violent and continued convulsion, and died in nine hours.

" *Inspection.* In the substance of the middle lobe of the left hemisphere of the brain, about the level of the lateral ventricle, there was a portion in a state of complete ramollissement, about an inch and a half in length, and an inch in its other dimensions, and the neighbouring parts appeared unusually vascular. The tuber annulare and pons Varolii were softer than usual, but otherwise healthy. No other morbid appearance could be discovered in the head, and all the other viscera were healthy." (P. 89.)

Dr. A. presumes, that in this case the sudden attack, so closely resembling the ordinary paralytic attack, must have been connected with the commencement of the inflammatory stage. The remarkable symptoms in the stomach, in the further progress of the disease, and the mode of its termination, render it altogether a case of much pathological value.

The following case shows the same morbid appearance, with a train of symptoms considerably different, but with a remarkable similarity in the mode of its termination.

" A gentleman, aged thirty-eight, during two years before his death, had suffered several epileptic attacks; from which, however, he had always speedily recovered. On the morning of 27th December, 1827, he was found in bed speechless and paralytic on the right side. He recovered his speech in the course of the day: the palsy continued in the usual manner, and after some time he began to recover a degree of motion of the parts. When he came to Edinburgh, about a month after the attack, he had recovered the use of his leg so far as to be able to walk once or twice across his room with much exertion; his arm was improved in a much less degree; his speech was distinct, but his mouth was considerably distorted, and his mind was somewhat impaired. He now consulted Dr. Thomson, and under the usual treatment he was progressively improving, so that at the end of another month he could walk along the streets to a considerable distance, though with a dragging motion of his leg, and could nearly raise his arm to his head. In the evening of 22d February he went to a supper party, and seemed remarkably well; but departed considerably from the abstemious regimen to which he had been previously restricted. About eight o'clock on the morning of the 23d, he was found in bed in a state of complete insensibility, accompanied by severe and general convulsion, which was strongest in the limbs of the right side. The face was much convulsed, the eyes rolling and insensible, the respiration laborious and convulsive. Bloodletting and the other usual means were actively employed, without any relief. The convulsion continued unabated in the state now described, when I saw him at eleven, and he died at two.

"*Inspection.* The brain externally was healthy, except some old adhesion of the membranes near the posterior part of the falx, and very trifling effusion under the arachnoid. The ventricles contained the usual very small quantity of fluid. On the outer side of the left ventricle, and separated from it by a thin partition of healthy cerebral substance, there was a defined portion in a state of complete and diffluent ramollissement. The portion thus affected was about an inch in depth; about half or three-fourths of an inch in diameter at the upper part; and became gradually narrower as it descended by the side of the ventricle, until it terminated almost in a point. There was considerable softening of part of the medulla oblongata, and the upper part of the spinal cord. No other vestige of disease could be discovered, on the most careful examination." (P. 91.)

Dr. A. does not attempt to offer any explanation of the symptoms in these two most remarkable cases, or to reconcile them with the old notions in regard to diseases of the brain. He gives them as facts, carefully ascertained and faithfully related, to be illustrated by further observations.

For the following case the author is indebted to Dr. Combe, of Leith It affords a good example of tubercular disease of the brain, and is interesting from the singular coincidence of the two forms of paralysis, on the opposite sides of the face: the one connected with the division of the portio dura, the other with the disease in the brain.

"A man, aged thirty-six, about a year before his death, had a tumor extirpated from behind the angle of the jaw, on the left side, and, immediately after the operation, paralysis took place on the left side of the face; in consequence of which his mouth was distorted to the opposite side in a most extraordinary degree. About six months after this he began to complain of headach and giddiness, which often gave him the appearance of intoxication; and after some time these symptoms were followed by impaired vision, occasional strabismus, and a considerable degree of deafness; and at last by drowsiness, coma, convulsions, and death. As these symptoms advanced, he became affected with numbness, and loss of power of the right side of the face, which increased very gradually. During the increase of this, the distortion of his mouth gradually diminished, and, for some time before his death, his countenance had become entirely symmetrical. Both sides of his face were now entirely paralytic; but with this difference, that on the right side the feeling was also lost, while on the left the feeling was entire.

"*Inspection.* In the centre of the middle lobe of the right hemisphere of the brain, there was a tubercle about an inch long, and three fourths of an inch in breadth. At its lower part it was attached to the cerebral substance, but the rest of it was detached, being surrounded with dark-coloured pus. In the vicinity there was increased vascularity, with softening of the cerebral substance." (P. 178.)

It is now a well-known fact that apoplexy, as well as insanity and various other diseases, the symptoms of which can only be referred to some cerebral disturbance, may prove fatal, and yet no morbid appearance whatever be detected in the brain, after the most careful examination. Dr. Abercrombie applies the term "simple apoplexy" to that variety of the disease in which the brain is apparently healthy. The phenomena of this disease appear fully to establish the important fact that there is a modification of apoplexy depending upon a cause of a temporary nature, without any real injury done to the substance of the brain; that the condition upon which this attack depends may be removed almost as speedily as it was induced; and that it may be fatal without leaving any morbid appearance in the brain. The following case occurred under the care of Dr. Duncan. A similar instance very recently occurred in our own practice.

"A man, aged fifty-four, of a plethoric habit and short necked, was admitted into the clinical ward on 30th May. He was in a state of nearly perfect coma, speechless, and with palsy of the right side to such an extent that even the intercostal muscles of that side did not act. The leg and arm of the left side were occasionally affected with convulsive motions. Breathing stertorous, deglutition much impaired; pulse seventy-four. The affection was of three days' standing, and had come on with vertigo, loss of vision, violent headach and vomiting.

"All the usual remedies were employed in the most judicious and active manner, without benefit. On the 1st of June there seemed to be a slight return of intelligence; but he soon relapsed into coma, and died on the 3d, without any change in the other symptoms.

"*Inspection.* A most minute and careful examination was made of the brain, without discovering any appearance of disease, except that the choroid plexus seemed rather darker than usual, and the basilar artery was diseased at one spot. By the side of the artery there was a spot of the cerebral substance, no larger than a barleycorn, which appeared somewhat softened; but even this Dr. Duncan considered as extremely doubtful." (P. 218.)

Upon this part of the subject, however, it behoves us not to be too presumptuous. With all our anatomical dexterity, disease may exist, although no vestige of it can be traced by the most skilful anatomist. Dr. Burrows, in discussing the physical causes of insanity, has very properly observed, that "we ought not to presume, because there are no visible marks of a morbid condition of the brain or its appendages, that therefore the whole are in a perfectly healthy state. Where is the anatomist who will dare maintain that a brain is free from disease or structural change, because, after the most minute inspection, he cannot discover any?"* It must be remembered that even in the elementary composition of the brain anatomists are not agreed.

The following case, for which Dr. A. is indebted to Dr. Macauley, is remarkable for the unusual rapidity of its fatal termination.

"A woman, aged forty-five, who had been for several years liable to headach, attended a crowded meeting on the evening of 25th June, 1829, and seemed in perfect health. Towards the conclusion of the meeting she uttered a loud and convulsive scream, and instantly fell down in a state of insensibility. She was immediately carried out, and was seen by Dr. Macauley, who happened to be present: he found her pale and totally insensible, and the pulse feeble; and within five minutes from the first seizure she was dead.

"*Inspection.* The integuments of the head were much loaded with blood. On removing the dura mater, there was a thin but very extensive appearance of extravasated blood, or rather ecchymosis, which covered nearly the whole surface of the brain. In the substance of the anterior lobe of the right hemisphere there was a coagulum of blood, the size of a large bean. All the other viscera were examined in the most accurate manner, but nothing was discovered, except a tubercle on the liver, and a small spot of ossification on the abdominal aorta." (P. 245.)

In the following instance a more extensive extravasation of blood, in all the ventricles, and along the whole course of the spinal cord, was detected, than had ever before been observed by Dr. Abercrombie. The case is remarkable from the period of life at which the affection took place, and from its similarity in the symptoms to one of the common inflammatory affections terminating by effusion.

"A boy, aged nine, previously in perfect health, awoke in the night of 18th May, 1829, complaining of headach; had vomiting and slight convulsion. On the 19th he was seen by Mr. W. Brown, who found him still complaining of headach, with occasional vomiting, but without any urgent symptom. Under the usual treatment the complaint seemed gradually to subside, and on the 25th he appeared to be entirely rccovered; but in the afternoon of that day he had a return of convulsion, and in the evening complained much of headach; pulse sixty-four.

26th and 27th, said he was better, but seemed drowsy. Pulse slow; bowels obstinate.

* Commentaries on Insanity, p. 70.

"28th, had two attacks of convulsion, the second of whichwas very severe and continued for several hours, affecting chiefly the left side of the body; pulse 130.

"On the 29th, he was again better; but from this time he became gradually more and more drowsy, and at last comatose, with squinting and occasional convulsive motions of the limbs, and he died on the 3d of June. His death was preceded by severe convulsion, of several hours' duration. I saw him, along with Mr. Brown, from the 29th.

"*Inspection.* The surface of the brain was healthy. The lateral ventricles were distended with dark bloody fluid, and each of them contained a mass of coagulated blood: that in the right was the size of a large walnut, the other smaller. The third and fourth ventricles were quite filled with coagulated blood in a very firm state; and, from the bottom of the fourth ventricle, the coagulum was traced outwards, and spread along the base of the brain and cerebellum, and around the medulla oblongata. The spinal canal being now laid open, the dura mater of the cord appeared remarkably distended, and the cord was found, through its whole extent, entirely enveloped by a very firm and uniform stratum of coagulated blood. The brain and cord were in their substance healthy, and the source of the hemorrhage could not be discovered." (P. 250.)

As a striking illustration of the nature of the symptoms which may exist with most extensive and remarkable organic disease of the brain, a case is added to the present edition, which fell under the observation of Dr. KELLIE.

"A medical gentleman, aged fifty-six, of a cultivated mind and temperate habits, had been for some time liable to various ailments, which his medical friends considered as in a great measure hypochondriacal. The most defined complaints were occasional uneasiness in the site of the frontal sinus, and a very peculiar feeling of numbness in the point of the thumb. But his general health appeared good, and he was able to enter into all the usual enjoyments of life, having retired from practice, till he was one day seized, while walking, with sudden sickness and faintness. These were followed by some headach, and an obvious difficulty of articulation, or rather a difficulty in finding the expression which he wished to make use of. He was now treated by bleeding and the other usual means; but this peculiar loss of the recollection of words continued and gradually increased, so that he had greater and greater difficulty in recollecting the words which he meant to employ, but he had no difficulty in pronouncing them. His understanding at this time was quite entire; his pulse varying from 80 to 112. He was nearly confined to the house, but out of bed during the day; and all the usual remedies were employed in the most assiduous manner. After he had gone on in this way for several weeks, he began to have slight distortion of the mouth, and complained of numbness of the right arm, and soon after of weakness of the right leg. These symptoms gradually increased to perfect hemiplegia; and about this time also he entirely lost his speech. He was now confined to bed, but without coma. He had the perfect use of his sight and hearing, and, as far as could be judged, his understanding was entire. He died with symptoms of bronchitis in the ninth week from the first attack.

"*Inspection.* The left hemisphere of the brain was found to be diseased throughout in a very singular manner. Some parts of the mass were indurated, others softened; and it presented a variety of colours, chiefly a rose colour, grey, and yellow; and the more diseased portions were highly vascular. In some places there were distinct insulated masses, enclosed in vascular cysts; these were generally indurated, but some were softened, and they were of a rose or flesh colour, passing into grey. The change from those parts which retained a natural appearance to these degenerated portions was abrupt, and marked by a rose-coloured line. These rose-coloured portions were chiefly in the parts nearest the surface; in the central parts this passed into the yellow or grey, and many portions were in a state of ramollissement. The whole left hemisphere, in fact, presented little else than a mass of concentric indurations and softenings of the various colours which have been mentioned. On the upper part of the hemisphere, the disease did not extend entirely to the surface of the convolutions; but at the base of the anterior and middle lobes it extended to the surface and at one place there was a well-defined spot of superficial ulceration, the size of a split pea." (P. 339.)

*Inflammation of the Substance of the Spinal Cord.* This important subject has not yet been investigated with that attention which it merits; but there is reason to believe that inflammation of the substance of the cord, like the corresponding affection of the brain, may terminate fatally in four different forms: viz. in the inflammatory stage, by ramollissement, by undefined suppuration, by abscess. Ramollissement of the cord sometimes occurs in a chronic form, in which it may go on for a considerable time, even for years, before it is fatal. There is generally in these cases some uneasiness in the back, with paralytic symptoms, beginning in a part of a limb, and in a slight degree, and advancing very gradually to confirmed palsy. The lower extremities are most commonly affected, but in some cases the arms only, and in others all the limbs. There is sometimes permanent contraction of the affected limbs, and sometimes there are spasmodic affections of them; and the disease may go on in this manner for years, and at last be fatal by ramollissement. This form is illustrated by the following case, which the author saw with Dr. Alison.

" A gentleman, aged forty-two, in October 1827, began to be affected with pain in the lower part of the back, stretching round the abdomen, and frequently shooting into the groins. After a short time this was succeeded by coldness and numbness of his feet, which gradually extended upwards with diminished power of motion, until, after several weeks, it terminated in perfect loss of motion of both lower extremities, with retention of urine. There was pain in some parts of the affected limbs, and in others a painful sensation of cold. This perfect loss of power continued five or six weeks, when, after a great deal of treatment by cupping, blistering, &c., he recovered a slight degree of motion, but no power of the bladder. He then began to be affected with spasms of the muscles of the back and abdomen, with a very uneasy sensation of tightness across the abdomen, and at times across the lower part of the thorax. The spasms occasionally assumed the characters of opisthotonos, and at one time he had almost incessant hiccup, which continued in a most violent degree for several days. After the employment of various antispasmodics, this subsided under the use of musk. During the course of these symptoms, he frequently complained of pain in various parts of the spine, at first in the lower part, and afterwards higher up; and the feeling of numbness extended gradually upwards, till it reached nearly the upper part of the dorsal region, and was felt in a very considerable degree along the sides of the thorax.

" After this he became liable to feverish attacks at night, terminating in the morning by very profuse perspiration; but this was strictly confined to the parts which were not palsied, and there never was the smallest moisture on the lower extremities. He had also, in the upper extremities, a frequent feeling of intense heat, while the lower continued cold and benumbed. During this time a considerable, but very imperfect, degree of motion continued in the lower extremities, but the bladder continued entirely paralytic.

" In April 1828, he went to the country, and at this time he had such a degree of motion as to walk a little on a smooth garden walk, leaning on two persons, or supported by crutches. But soon after this, he began to complain of pain in the head. It occurred in irregular paroxysms, and was often referred to a small defined spot, on various parts, especially behind the ear, and sometimes to the tip of the ear. This pain seemed to abate under the use of arsenic; but soon returned, and became more fixed and permanent, and the palsy of the limbs again increased. After an absence of about two months, he returned to town in the beginning of July. At this time the headach was severe, and the power of the limbs so much impaired that he was entirely confined to bed. In a few days after his return, the right arm became paralytic, and his speech considerably impaired. After a day or two these symptoms rather subsided, but in the following night he became comatose, and died in the afternoon. There never was complete loss of sensation of the affected limbs: he had only complained of it occasionally at particular spots, and of a general feeling of numbness and coldness.

"*Inspection.* There were some scales of bone attached loosely to the inner surface of the dura mater of the spinal cord. The whole cord was of a pale rose colour, and in a state of complete ramollissement through its whole extent, being in every part entirely diffluent. The medulla oblongata was tolerably healthy, except a slight degree of softening on its anterior part; and there was also a degree of softening on the tuber annulare, which seemed to involve the origin of the fifth nerve. Beyond this the ramollissement became again more decided, extending along the crura cerebri and cerebelli, and considerably into the substance of the brain, at the part adjoining the crura. The brain in other respects was healthy, and there was no effusion in the ventricles." (P. 364.)

It is difficult to trace the precise nature and progress of the affection of the cord, when the disease advances in so gradual a manner as in this case, and terminates in disorganization so complete and extensive. In tracing the history of the analogous disease of the brain, Dr. A. suggests, upon very strong grounds, that it is originally an inflammatory affection of a low chronic character, seated in a small part of the cerebral substance; that it may continue for a considerable time in the state of simple inflammation, and then subside; or that it may terminate by a permanent change in the structure of the part, generally with some degree of inflammation.

To the chapter on the *Affections of the Bones of the Spine* a case is added, which shows disease of the processus dentatus, complicated with a new formation, presenting the characters of fungus hæmatodes.

"A gentleman, aged twenty-two, of a scrofulous habit, in the early part of his life had suffered amputation on account of a disease of the knee, and afterwards was liable to pectoral complaints with hæmoptysis. In the beginning of the year 1828, he began to complain of pain and stiffness of the neck, referred chiefly to the left side of it, and much increased by the motion of the head. The pain sometimes extended into the larynx, and backwards towards the scapula. After considerable relief from repeated blistering, &c. the symptoms returned, accompanied by loss of appetite, frequent pulse, and night perspirations; and soon after this he became affected with difficult deglutition, some dyspnœa and hoarseness. There was now also severe fixed pain referred to the back of the head, and much increased by the motion of the parts; so that he was obliged to support his head with both his hands when he had occasion to make any change of his posture. He was next affected with paralysis of the tongue and the upper eyelid of the left side. On 16th January, 1829, he was seized with paralysis of the left arm; and two days after the right was affected in the same manner. He had then great pain and difficulty in passing urine, with obstinacy of the bowels, which nothing could overcome. On the 29th, the lower extremities became paralytic, and he died on the 31st, having suffered greatly on the day on which he died, from difficult breathing.

" *Inspection.* All the external parts of the neck, the pharynx, &c. were healthy, and no disease was discovered in any of the vertebræ in their external aspect. The brain and cerebellum were healthy, except some increase of vascularity. Within the foramen magnum, and attached to the inner surface of the dura mater, at its anterior and lateral parts, there was a spongy tumor of a greyish yellow colour, which, when cut into, presented a variegated structure, resembling fungus hæmatodes. The processus dentatus was rough and carious on its surface, and it was so much elongated as to project half an inch into the cavity of the cranium. Its ligaments also were partially destroyed, so as evidently to allow it to encroach upon the area of the spinal canal, and to compress the cord. The spinal cord at the upper part was flattened, but not materially altered in its texture." (P. 408.)

In regard to the treatment of the diseases of the spinal cord, Dr. A. does not enter into any long details, as it must be regulated by the same principles as the corresponding affections of the brain. In the more acute affections, we must, of course, rely chiefly on free general and topical bleeding, assisted by blistering, purgatives, and the other usual auxiliaries. When the affection is in a more chronic form, the treatment will consist chiefly in local applications, as topical bleeding, blistering, and issues, aided by the horizontal posture. In the earlier stages of such affections, Dr. A. thinks the most satisfactory treatment, after free topical bleeding, is by a succession of blisters, applied first on one side of the spine and then on the other, in quick succession, and repeated in this manner to a considerable number. In some of the cases great benefit is also obtained from continued moderate purging.

There are certain obscure and anomalous affections which present many of the characters of disease of the spinal cord, though their termination, in general, is more favorable. These affections assume a great variety of characters, and the nature of them is very obscure. The most common symptoms are various spasmodic affections of the limbs, or of the muscles of the back, sometimes resembling chorea, or even tetanus; and various degrees of weakness of the lower extremities, sometimes amounting to complete paralysis, which is often accompanied by remarkable spasmodic affections of the paralytic limbs. There is generally a great feeling of weakness in the back, and frequently pain, which is sometimes confined to one part, but more commonly extends in a greater or less degree along the whole of the spine. Various affections of the breathing likewise occur, sometimes with attacks of palpitation, and different uneasy feelings in the stomach and bowels.

It is, indeed, difficult to say what treatment has any decided control over those affections; but the remedies which appear to be most beneficial are free and regular purging, or a combination of tonics and antispasmodics, with small doses of purgatives; strong friction; cold sponging, or shower bath; and blistering on the spine. These affections commonly pass off without leaving any bad consequences, sometimes very suddenly, and without any cause to which their removal can be ascribed.

Such attacks occur almost entirely in females, chiefly those of the higher rank, and are generally tedious and untractable. One of the most remarkable features is the connexion which they have, even in their most aggravated forms, with the state of menstruation. The following case, which is not contained in the first edition, illustrates this fact in a striking manner, and at the same time exemplifies some of the various forms which are assumed by those singular affections.

"A lady, now aged twenty-four, in the year 1823 was first affected with numbness and partial loss of power of the right arm and leg, and some time after had slight difficulty of articulation. These symptoms subsided under the usual treatment, and returned after some months, when they affected the legs and arms of both sides, and had more of the characters of chorea. After another interval of several months, she became liable to attacks of blindness, which were occasioned by a falling down of the upper eyelids, so that she could not raise them; and, when they were raised by the hand, the eyes were found to be distorted upwards. These attacks generally continued for several weeks at a time, and were relieved by cupping on the temples.

"With these symptoms the two first years of her illness passed. In the third year she was affected with convulsive action of the muscles of the back, and involuntary twitches of the legs and arms, producing convulsive motions of the whole body, which it is impossible to describe. These were much increased by touching her, especially on any part of her back; also by laying her upon her back, or even by approaching her as if with the intention of touching her. At one time there was difficulty of deglutition, so that attempts to swallow produced spasms resembling tetanus. At other times, after lying for a considerable time quiet, she would in an instant throw her whole body into a kind of convulsive spring, by which she was thrown entirely out of bed; and in the same manner, while sitting or lying on the floor, she would throw herself into bed, or would leap on the top of a wardrobe fully five feet high. During the whole of these symptoms her mind continued entire, and the only account she could give of her extravagance was, a secret impulse which she could not resist.

" After a considerable time these paroxysms ceased, and she was then affected with convulsive motions of the muscles of the upper part of the back and the neck, producing a constant rotatory motion of the head. This sometimes continued without interruption night and day for several weeks together; and, if the head or neck were touched, the motion was increased to a most extraordinary degree of rapidity. During the attacks she could not sleep except in the sitting posture, the motion continuing during this imperfect sleep, though in a more moderate degree; but if she happened to slip down, so that her head touched the pillow, she instantly awoke with a severe convulsive start, and the motion was increased to the greatest degree of rapidity. These paroxysms were relieved by nothing but cupping on the temples to the extent of ten or twelve ounces, when the affection ceased in an instant with a general convulsive start of the whole body. She was then immediately well, got up, and was able to walk about in good health for several weeks, when the symptoms returned, and required a repetition of the same treatment. Sometimes, from the violence of the motion of the head, it was impossible to cup her on the temple. In this case the cupping was applied first on the back; and by this the motion was so far moderated as to allow it to be applied on the temple, without which the paroxysm was never removed. Bleeding from the arm to the extent of faintness only moderated it for a time, but did not remove it. Another very singular feature of the affection was, that it subsided fully only when it went off in an instant with a sudden convulsive start of the whole body: when it subsided gradually, as under the influence of large bleeding, it returned as soon as the faintness from the bleeding was removed.

" The affection went on in this manner, with intervals of tolerable health of a few weeks' duration, for about four years, besides the two years formerly mentioned. The longest interval was one of about three months; but even during these intervals various convulsive motions were excited by slight causes. Menstruation was all along extremely irregular and very scanty, and the bowels were torpid. She was of a pale and bloodless aspect, from the frequent bleedings, but not reduced in flesh. I saw her only at an advanced period of the disease, along with Mr. Gillespie, who had watched her through its whole progress, and by whom every variety of treatment had been employed with the utmost assiduity.

" At last, in the spring of 1829, we found her under a severe paroxysm of the rotatory motion of the head; when it was determined to allow the attack to take its course, and to direct our attention entirely to the menstruation. With this view she began to take three grains of the sulphate of iron three times a day, with two grains of Barbadoes aloes; the aloes being afterwards diminished according to the state of the bowels. She went on with this for nearly three weeks; the convulsive motion of the head continuing without intermission night and day. At length, in the middle of the night, the paroxysm ceased in an instant, with the same kind of convulsive start of the whole body with which it used to cease after cupping. At the same time menstruation took place in a more full and healthy manner than it had done for many years. She has continued from that time free from complaint, and able to walk several miles, and menstruation has occurred at the regular periods, and in a full and healthy manner." (P. 328.)

We have now drawn very largely from the additional cases with which Dr. Abercrombie has enriched the present edition of his very excellent work. The chapter on *Diseases of the Nerves* is also augmented by many interesting facts and observations, chiefly in reference to the practical importance and application of the physiological experiments and discoveries of BELL, SHAW, and MAYO.

But few physicians can have enjoyed the opportunities which have fallen to the lot of Dr. Abercrombie, of improving our pathological knowledge; and it would be unjust not to declare that he has turned his experience to the best account. The details of some of the cases might, perhaps, be compressed with advantage, and without any danger of obscuring their important practical features. We cannot but be struck with the great superiority of such works as those for which we are indebted to Dr. Abercrombie over the abstract speculations as to the proximate causes of disease, in which some pathological writers delight to dwell. The numerous and faithful records of disease given to the profession by Dr. A. can never lose their value, either to the practitioner or student. They are alike creditable to his industry and to his ability.

---

*Elements of General Anatomy, containing an Outline of the Organization of the Human Body.* By R. D. GRAINGER, Lecturer on Anatomy and Physiology.—8vo. pp. 526. Highley, London, 1829.

THE term "general anatomy," as it is employed by modern writers, is but of recent introduction. It designates that branch of anatomy which has for its object the investigation of the various tissues of the body, and is employed for this purpose by BICHAT, BECLARD, and MECKEL. Mr.

Grainger correctly observes, that in some respects this expression is objectionable; for, in its strict acceptation, it comprehends every thing that relates to the science of organization; but custom has sanctioned the use of it in this more limited meaning. It must be confessed that we are greatly, if not chiefly, indebted to the continental writers, amongst whom Bichat claims the most distinguished rank, for the great additional light that has, within the last few years, been thrown upon this most important branch of anatomical knowledge.

It is as essentially necessary for the medical and surgical student to possess a perfect acquaintance with the intimate structure of the various textures of the body, as that he should be correctly versed in mere descriptive anatomy.

When Mr. Grainger commenced the present volume, we had no work exclusively devoted to the consideration of the texture of the different parts of the human body. After he had collected his materials, the work of Dr. Craigie,* of Edinburgh, appeared, to the excellence of which we have offered our meed of approbation. The plan of these works is, however, very different; and, even had they been entirely similar, much benefit would have been conferred upon the student by the labours of both writers.

The subject is too intricate and comprehensive to be exhausted by any single author, whatever may be his ability. It must even be still confessed that an ample field remains for investigation. We are yet to learn many important facts connected with "general anatomy," to remove many doubts, and to reconcile many conflicting opinions. An additional work upon the subject, then, cannot be deemed superfluous, and it could not have been in better hands than Mr. Grainger's, who is well known to the profession to possess those first and most essential requisites for increasing our stock of knowledge, talent and determined industry.

The object of the present work is to convey a concise, and at the same time a comprehensive, account of the several substances which form the human body. To the description of the different tissues some observations on their uses are added, for the purpose of showing how admirably each structure is adapted to the functions it is destined to fulfil.

It would be impossible to compress within the limits to which we are confined an analysis of each division of a work which comprehends so many subjects. We can but

* Elements of General and Pathological Anatomy.

give a general view of the contents, and then offer our opinion of the claim which the work possesses to the attention of the profession.

The introductory part of the volume embraces many very interesting points of discussion. It commences by the description of the distinctive characters which separate inorganic or mineral bodies from organic or living bodies. These two great divisions are distinguished from each other by certain properties, which are determined and invariable.

"The composition of inorganic substances is characterised by the following circumstances: 1. Its homogeneousness. 2. The independence of its molecules, each of which is capable of existing independently of the others. 3. The simplicity of its chemical properties. 4. Its peculiarity of constitution, consisting of gaseous, liquid, or solid substances, and never exhibiting a union of fluid and solid parts. 5. Its capability of being decomposed and recomposed.

"The composition of living beings exhibits, in all the preceding circumstances a striking contrast. 1. The living body is heterogeneous, consisting of dissimilar parts. 2. Its constituent molecules have a mutual and necessary relation to each other, and, consequently, cannot preserve an independent existence. 3. Its elementary substances are numerous, and combined in varying proportions. 4. The fluid and solid parts of which it is composed are intimately combined together, and mutually influence each other. 5. It is capable of decomposition, but totally incapable of artificial recomposition." (P. 1.)

The origin or first formation of minerals results from the operation of external circumstances: thus they are produced by the separation of the particles which compose other minerals, or by the combination of elementary substances, which are united in virtue of their chemical affinities. Organised bodies, on the contrary, owe their origin to an internal operation, which is termed generation: in this process a substance, called a germ, is attached for a certain period to another similar being, from which it is subsequently detached, and then enjoys a separate and independent existence. The primitive attachment of every living body to a similar being, which bears to it the relation of a parent, is probably a rule without an exception. It was, however, supposed by the ancients that organised bodies might be formed like minerals, by the operation of the general laws of matter. The curious discoveries that have been made concerning the existence of the numerous species of infusory animalculæ, have induced several modern physiologists, among whom may be mentioned M. Lamarck, to revive the doctrine of equivocal or spontaneous generation. The extreme minuteness of these animalcules renders it impossible to determine, by examination, whether they are formed simply by the combination of the surrounding elementary molecules, or from ova which had been previously deposited in the water used for the experiment. The probabilities are decidedly in favor of the latter opinion.

The line of demarcation is by no means so positively drawn between vegetables and animals, as between inorganic and organic bodies; for both the two former great classes participate in many of those properties which are the most essential attributes of organised bodies. Still the distinctive marks of plants and animals are numerous and decisive, and are thus enumerated by the author:

"Vegetables are fixed to the earth, have no perceptible motion.
Have probably an obscure kind of sensibility, without consciousness.
Are composed of few elements, have much solid matter, and have carbon for their base.
Resist decomposition.
Are nourished by external absorption.

"Animals move upon the surface of the earth.
Have sensibility accompanied by consciousness.
Are composed of many elements, have a large quantity of fluids, and have azote for their base.
Are readily decomposed.
Are nourished by digestion and internal absorption."

Having pointed out these necessary distinctions between the different material substances which compose the globe of the earth, or which exist upon its surface, Mr. Grainger proceeds to describe the general structure of the human body, and then considers each part separately which enters into the composition of the animal frame. An excellent and clear description is first given of that most extensive and important of all the tissues, the cellular membrane. The serous and mucous membranes are next described at considerable length. The vascular, lymphatic, cartilaginous, fibrous, fibro cartilaginous, and osseous systems, are also dwelt upon with due attention; and the various sections which are occupied in pointing out the many and interesting phenomena belonging to them will be studied with much advantage.

Mr. Grainger is opposed to Dr. Craigie upon the subject of the contractility of the capillary vessels. In our notice of Dr. Craigie's work,* we contended that the experiments of Hunter, Wilson Philip, Thomson, and Hastings, satisfactorily proved the contractile power of these vessels. Dr. C. is of a different opinion.† As the subject is of much importance, not merely in a physiological point of view, but as our pathological notions and treatment of disease must be frequently modified by the ideas we entertain of the power of the capillaries, we extract the observations which Mr. Grainger offers. In our opinion, he takes a correct view of the subject.

"The actions of the capillary vessels are essential to the production of most of those operations which are required for the support of life. The opinions of physiologists are, in the present day, divided as to the existence of an active contractile power in these small blood-vessels; and when the great importance of the question is considered, we cannot be surprised at the number of writers who have engaged in the controversy. According to some authorities, the capillaries are merely passive tubes, which are not provided with irritability. Others, on the contrary, contend that they have a contractile power, which enables them to carry on the circulation quite independently of the heart: this power was called by Bichat, insensible organic contractility. Lastly, many excellent physiologists suppose that the capillary circulation is influenced by the action of the heart; but that the small vessels have themselves an active force, which enables them to assist in propelling their contents, and which may be exercised independently of the heart. As it would be difficult to reduce within the limits of this work the numerous considerations that are connected with this most comprehensive subject, I shall confine myself to pointing out some facts which appear to prove the correctness of the last opinion.

"It has been already shown that the propulsive power of the ventricle extends to the venous system. Now, as it is evident that that power must have previously acted on the capillaries, I conceive the first part of the position must be admitted, viz. that the capillary circulation is influenced by the action of the heart.

"The second part of this opinion may be substantiated by observing certain phenomena which occur in the human body, and by the results of experiments performed on the lower animals. It is well known that local action frequently takes place, by which blood, and other fluids, are determined towards individual parts of the body, without the heart's action or the general circulation being in the least affected. Mental emotion is often the exciting cause of the great local accumulation. Thus, shame causes blushing; voluptuous ideas produce erection; and the sight of food

* London Medical and Physical Journal, March 1829, p. 254.

† CRAIGIE's Elements of General and Pathological Anatomy, p. 142.

excites, in a hungry person, a flow of saliva. At other times local action may be produced by mechanical irritation: for example, titillation causes erection of the nipple, a particle of iron renders the vessels of the conjunctiva turgid, &c. Again, in local inflammation, the activity of the small arteries is increased, although the condition of the heart is not affected. It has, indeed, been implied, by an author of great excellence, (Dr. Charles Parry,) that, in similar instances to those above mentioned, a prior action occurs in the heart, which is the occasion of the local distention. But, if this supposition were founded in truth, which there is great reason to think it is not, it would not explain the phenomena which are observed. Such an increase in the power of the heart would certainly accelerate the general circulation; but we cannot conceive how it could influence the flow of blood in any particular set of vessels, unless those vessels had themselves a local source of action.

"The important experiments which have been performed by Dr. W. Philip, Dr. Thompson, and Dr. Hastings, show, in a still more striking manner, the contractile power of the capillaries. Thus, the circulation continues in them after a ligature has been tightly bound around the leg of a frog; after the great vessels of the heart have been all tied; nay, more, after the heart itself has been removed, or has long ceased to act. The contractility of the capillary vessels may also be excited by the direct application of stimulants, without the action of the neighbouring arteries being at all influenced.

"The conclusion to which the preceding facts lead is, that the small blood-vessels are provided with an active and independent power, which materially assists the heart in propelling the blood onwards to the veins.

"The functions of the capillaries are much more important than those which are exercised in the other parts of the vascular system. The arteries and the veins are, in fact, entirely subservient to the action of these vessels: the former, by bringing the fluid material, the blood, which is destined to fulfil certain uses, and to undergo certain changes, as it traverses the capillary system; the latter, by returning the altered blood after the necessary processes have been accomplished. The general capillaries, or those placed between the termination of the aortic arteries and the origin of the common veins, are the agents which effect the vital functions of secretion and nutrition, in the completion of which, the blood experiences such changes, that it becomes deteriorated and unfitted for the purposes of the economy. The capillaries of the lungs are connected with equally important processes; for, in these vessels, the blood is renovated by being freed from the noxious principles it had acquired in the general circulation; and in the same tubes the chyle and the lymph are assimilated with the nutritious fluid.

"It is very difficult, or more correctly speaking, impossible, in the present state of our knowledge, to decide on the real nature of the phenomena which occur in the capillary vessels. They appear, however, to be of a mixed character, and to depend on the conjoined operation of mechanical, chemical, and vital actions." (P. 301.)

The section on the *reparation of fractured bones* is highly instructive, and gives a concise account of the opinions of different celebrated writers upon this important and practical subject. The older anatomists attributed the production of *callus* (by which term is understood the substance connecting the broken ends,) to the exudation of an osseous juice from the surface of the fracture, which, gradually acquiring consistence and hardness, at length united and soldered together the fragments. This opinion reigned in the schools till Duhamel, towards the middle of the last century, opposed it, by publishing the results of his experiments. He found that, shortly after a bone was broken, the periosteum, being thickened and inflamed, was glued to its outward surface, and that in a few days later, when the swelling of the membrane was increased, its internal layers were converted into cartilage, and ultimately into bone. From the result of his extended inquiries, Duhamel concluded that the periosteum was the part principally concerned in the reparation of fracture. The statements of Duhamel accord in many respects with the latest and best accounts which have been given of the formation of callus.

"The ingenious and exact investigations of Mr. Howship have determined most of the disputed points connected with this question, and have also explained several of the causes that have been most fertile in producing the diversity of opinion which has so long existed among physiologists. These facts perfectly accord with those ascertained by Dupuytren and Breschet, and therefore they may be received with the greater confidence. There is so much resemblance in the conclusions of these observers, that it would be an invidious task to apportion the credit that each individual deserves; but it is due to the character of the English experimentalist to state, that, although the papers of Dupuytren and Breschet had the priority as to publication, there is no reason to suppose that they were known to Mr. Howship previous to the appearance of his memoir.

"We learn from the united labours of these physiologists, that the first effect produced by a fracture is the effusion and coagulation of a large quantity of blood, which is derived from the lacerated vessels of the bone, of the periosteum, and even of the surrounding structures; the quantity being proportioned to the violence of the accident. This coagulum surrounds and unequally adheres to the broken ends, and a portion of it is deposited within the opening of the medullary canal, with which it is connected; the periosteum is also completely charged with the effused blood. The vascularity of the medullary membrane in the seat of the fracture is greatly increased, producing a bright vermilion-coloured surface, in which, with the aid of the microscope, the vessels may be seen quite entire. In the femur of the rabbit, no arteries could be detected passing into the coagulum, as late as the fifth day after the fracture. In a few days afterwards, the colouring part being removed, the extravasated blood becomes pale: at this period the periosteum is greatly thickened, and has been even found a quarter of an inch in thickness; it has a transparent pearly hue, and assumes by degrees the characters of true cartilage. The swelling and firmness of the periosteum, and of the surrounding parts, appear to be a provision of nature to guard against the least disturbance or motion between the fractured ends during the act of union.

"A deposit of osseous matter is made in the cartilaginous periosteum, and also in the coagulum that closes the medullary cavity: it is worthy of remark that, in the latter part, the deposition, as in the original formation of bone, advances from the circumference towards the centre of the coagulum. Mr. Howship found that the fractured ends of the femur of a rabbit were covered, on the twenty-third day, with a considerable quantity of new bone, clothed externally with a well-injected membrane or periosteum. After the bone is firmly united by the callus which is deposited between its extremities, the thickened periosteum and the surrounding soft parts are gradually restored to their original condition; and the ossific matter, which had been temporarily secreted so as to form an external callus, is slowly removed by the action of the absorbents.

"Baron Dupuytren concludes from his investigations, that there are two distinct processes in the union of fracture, or rather that a double callus is formed. The first, which he calls provisional callus, 'cal provisoire,' is formed by a deposition either in the periosteum alone, or in that membrane and in the cellular, and even in the muscular tissues. This secretion forms a kind of clasp which surrounds the broken ends, and also adheres to them. At this period the surfaces of the fracture are not united, but they are surrounded and supported by the new formation. In the space of four or five months, provided there has been perfect coaptation, and there is no irregularity around the fragments, the osseous substance which has been temporarily produced decreases in quantity, and the periosteum and the cellular tissue return to their original condition; lastly, at about the eighth month, the definitive or permanent callus is formed, by which the surfaces of the fracture are firmly joined." (P. 415.)

The tenth and eleventh chapters contain very complete descriptions of the muscular and nervous systems. An account is given of the arguments and experiments of Mr. Bell upon those nerves which he terms *superadded*, or *respiratory* nerves, and of the ingenious comments upon these experiments by Mr. Mayo,* which tend to throw much doubt upon some parts of Mr. Bell's celebrated theory. Upon this subject we shall enter at some length in our succeeding Number.

In concluding his observations upon the functions of the ganglionic system of nerves, Mr. Grainger alludes to an error, which, having received support from very high authorities, has had an injurious effect upon our inquiries concerning the functions of the nervous system. "I allude to the doctrine according to which the ganglia are provided to cut off the parts they supply with nerves from all connexion with the brain. The fallacy of this opinion has been demonstrated by Dr. Wilson Philip, as far as the ganglions of the great sympathetic are concerned; and in an equally satisfactory manner by Mr. Bell, with respect to the ganglions of spinal nerves."

The general sketch we have given of Mr. Grainger's work will be sufficient to show the subjects it embraces. It contains abundance of information, which it behoves the student to make himself thoroughly acquainted with; for, without a perfect knowledge of the intimate structure of every part of the human body, it is impossible he can see clearly through many of the most important morbid processes with which he will have to contend when he enters upon the practical duties of his profession.

* See Anat. and Phys. Comment. part i. p. 107; and Outlines of Physiology, by Herbert Mayo.

